# Robust Food Anticipatory Activity in BMAL1-Deficient Mice

**DOI:** 10.1371/journal.pone.0004860

**Published:** 2009-03-20

**Authors:** Julie S. Pendergast, Wataru Nakamura, Rio C. Friday, Fumiyuki Hatanaka, Toru Takumi, Shin Yamazaki

**Affiliations:** 1 Department of Biological Sciences, Vanderbilt University, Nashville, Tennessee, United States of America; 2 Osaka Bioscience Institute, Suita, Osaka, Japan; University of Queensland, Australia

## Abstract

Food availability is a potent environmental cue that directs circadian locomotor activity in rodents. Even though nocturnal rodents prefer to forage at night, daytime food anticipatory activity (FAA) is observed prior to short meals presented at a scheduled time of day. Under this restricted feeding regimen, rodents exhibit two distinct bouts of activity, a nocturnal activity rhythm that is entrained to the light-dark cycle and controlled by the master clock in the suprachiasmatic nuclei (SCN) and a daytime bout of activity that is phase-locked to mealtime. FAA also occurs during food deprivation, suggesting that a food-entrainable oscillator (FEO) keeps time in the absence of scheduled feeding. Previous studies have demonstrated that the FEO is anatomically distinct from the SCN and that FAA is observed in mice lacking some circadian genes essential for timekeeping in the SCN. In the current study, we optimized the conditions for examining FAA during restricted feeding and food deprivation in mice lacking functional BMAL1, which is critical for circadian rhythm generation in the SCN. We found that BMAL1-deficient mice displayed FAA during restricted feeding in 12hr light:12hr dark (12L:12D) and 18L:6D lighting cycles, but distinct activity during food deprivation was observed only in 18L:6D. While BMAL1-deficient mice also exhibited robust FAA during restricted feeding in constant darkness, mice were hyperactive during food deprivation so it was not clear that FAA consistently occurred at the time of previously scheduled food availability. Taken together, our findings suggest that optimization of experimental conditions such as photoperiod may be necessary to visualize FAA in genetically modified mice. Furthermore, the expression of FAA may be possible without a circadian oscillator that depends on BMAL1.

## Introduction

For an animal to flourish in its niche, food-seeking behavior must be spatially and temporally optimized. One such temporal component of foraging is anticipatory activity prior to food availability [1], [2], [3]. When food is restricted to a single recurring daytime meal, two distinct bouts of activity are observed in nocturnal rodents: a nighttime activity rhythm that free-runs in constant lighting conditions and a daytime bout of activity that commences prior to mealtime [4], [5]. Food anticipatory activity (FAA) is robust during restricted feeding of rodents with lesions of the suprachiasmatic nuclei (SCN) [4], [6], [7], [8], but the free-running rhythm of nocturnal activity is abolished [9], [10], suggesting that the light-entrainable oscillator (LEO) in the SCN is anatomically distinct from the food-entrainable oscillator (FEO).

The identification of the anatomical substrate of the LEO has fostered meticulous analysis of entrainment, the molecular mechanisms of timekeeping, and the output pathways of this circadian pacemaker. In contrast, the mechanisms underlying entrainment to food and timekeeping in the FEO remain enigmatic, which may be partially attributable to unsuccessful attempts to identify the locus of the FEO [2], [11]. Two putative timing mechanisms have been purported to account for FAA [2]. First, FAA could be the output of an interval timer, such that when the depletion of nutrients reaches a critical threshold, anticipatory activity ensues. The interval timer could also measure the time between a meal and the phase of the LEO or another circadian oscillator. Alternatively, the second model proposes that a self-sustained oscillator that is anatomically distinct from the SCN, and persists in the absence of restricted feeding, controls FAA.

Multiple lines of evidence suggest that the FEO is not an interval or hourglass timer that measures the depletion of nutrients. For example, even though FAA disappears during *ad libitum* feeding following a restricted feeding regimen, it reappears at approximately the same time of prior entrainment to feeding during food deprivation, suggesting that the FEO continues to run during *ad libitum* feeding [2]. In addition, entrainment of FAA is limited to periods between 20 and 28 hours in non-SCN lesioned rats [12], [13]. This limited range of entrainment is similar to the SCN, which is theoretically limited by the maximum phase shift that can occur during a single period [14], [15]. These data suggest that FAA relies on an oscillator since an hourglass timer should be functional regardless of the interval between meals. Finally, transient cycles of FAA occur after phase shifts of mealtime [16], [17]. Transient cycles of nocturnal activity are also observed when the LD cycle and the underlying LEO are phase-shifted [15], [18].

Even if the FEO is an oscillator, it may not operate in the same way as circadian oscillators in the SCN and periphery. The molecular mechanism of endogenous rhythm generation in the SCN and in peripheral oscillators is modeled as interlocking positive and negative transcriptional and translational feedback loops of circadian gene expression [19]. According to this model, the transcription of three *Period* homologs (*Per1, 2, 3*) and two *Cryptochrome* genes (*Cry 1, 2*) is activated by CLOCK and BMAL1 protein heterodimers. As PER and CRY protein complexes accumulate, they repress their own transcription. A secondary feedback loop composed of REV-ERbα and RORα drives the rhythmic transcription of *Bmal1*. BMAL1 is critically important for the function of the circadian oscillator in the SCN since BMAL1-deficient mice in constant darkness have arrhythmic locomotor activity and circadian gene expression in the SCN [20]. NPAS2 is a transcription factor that is expressed predominantly in the forebrain and is highly related in primary amino acid sequence to CLOCK [21], [22], [23]. Similar to CLOCK, NPAS2 binds to BMAL1 and is suppressed by the CRY proteins [24], [25], [26]. Although Garcia et al. showed that NPAS2 is not expressed in the SCN [21], DeBruyne et al. demonstrated that wheel-running running behavior is rhythmic when the *Clock* gene is nonfunctional, but arrhythmic in mice deficient in both NPAS2 and CLOCK, suggesting that NPAS2 is expressed in the SCN and is functionally redundant with CLOCK [27].

If the LEO and FEO use the same molecular mechanisms to keep time, then they should respond similarly to biochemical and genetic alterations of the clock. For example, previous studies have demonstrated that PERIOD2-deficient mice have shorter free-running periods or arrhythmic nocturnal activity [28], [29] and do not display food anticipatory activity [30], suggesting that PER2 is a fundamental component of the timekeeping mechanisms in both the LEO and FEO. A recent study also reported that BMAL1-deficient mice, that are arrhythmic in constant darkness (DD) [20], do not anticipate daily meals [31]. In contrast, multiple lines of evidence suggest that the LEO and FEO use distinct timekeeping mechanisms. First, homozygous *Clock* mutant mice, which express a dominant negative form of CLOCK [32], show FAA during restricted feeding and food deprivation in DD [33] when nocturnal activity is arrhythmic [34]. Second, *Cry1/Cry2* double knockout mice exhibit FAA, albeit abnormal, in DD [35] when nocturnal activity is arrhythmic [36]. Third, *Npas2*-deficient mice display mostly normal wheel-running activity [27] but take longer to acquire FAA [37], suggesting that NPAS2 is not essential for timekeeping in the LEO, but is important for acquisition of food entrainment in the FEO. Finally, deuterium oxide administered to rats lengthens the free-running period of nocturnal activity (LEO), but does not affect FAA [38].

The majority of studies investigating FAA have been performed in rats because of their ability to adapt to restricted feeding regimens and because they can survive several days of food deprivation. However, the development of mice with genetically altered circadian mechanisms makes them an attractive species for investigating the molecular components of the FEO. While studies of FAA in mice have become more prevalent, the conditions which foster high-resolution expression of FAA in mice have not been optimized. The use of sub-optimal experimental conditions may have led to erroneous conclusions that FAA is not observed in circadian mutant mice. To circumvent this problem, we optimized the conditions for examining FAA in BMAL1-deficient mice. By varying the experimental conditions, we found that BMAL1-deficient mice exhibited robust FAA during restricted feeding, suggesting that the expression of FAA may not require a BMAL1-dependent circadian oscillator.

## Results

### Optimization of food deprivation

**Figure 1:**
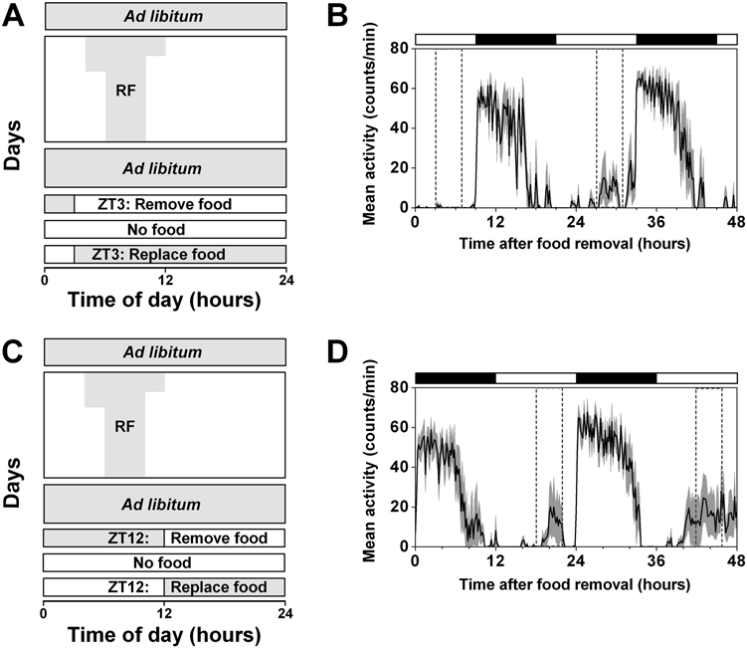
The timing of food deprivation affects food anticipatory activity (FAA). Diagrams of restricted feeding protocols when mice are food deprived starting at ZT3 (A) or ZT12 (C). Wildtype C57BL/6J mice were fed *ad libitum* and then food availability was gradually reduced (RF) from 8 hrs/day to 6 hrs/day. Then mice were fed 4 hrs/day (from ZT6 to ZT10) for at least 9 days and then provided food *ad libitum* for 6 days. The time when food was available is indicated by gray shading in A and C. On the seventh day of *ad libitum* feeding, food was removed at either ZT3 (A, B) or ZT 12 (C, D). Food was replaced 48 hrs later. Group average activity profiles for mice that began fasting at ZT3 (B; n = 4) or ZT12 (D; n = 5) were generated by averaging the number of wheel revolutions per 10-minute bin (black line) and were plotted relative to the light-dark cycle where ZT0 is the time of lights on and ZT12 is lights off. The SEM, which represents the variability among mice, is shown in dark gray shading. The time of previously scheduled food availability is indicated by dotted lines in the activity profiles. The light-dark cycle is indicated by the white and black bars, respectively.

After restricted feeding and *ad libitum* access to food, rats exhibit activity at the approximate time of previous food availability during food deprivation [2]. FAA disappears during *ad libitum* feeding between restricted feeding and food deprivation, suggesting that rats must be sufficiently “hungry” or that some resource must be depleted beyond a critical level in order to observe FAA. FAA during food deprivation is easily observed in rats since they readily survive several days of fasting. In contrast, the length of food deprivation in mice must be limited to about 48 to 60 hours to ensure their survival. Therefore, the timing of food deprivation relative to the previous time of food availability may be critical for observing FAA during fasting. If food is removed only a few hours before the time of previous food availability, then mice may not be “hungry” enough to express FAA on the first day of food deprivation. To test this hypothesis, wildtype mice were fed for 4 hrs/day, from zeitgeber time (ZT) 6 to ZT10, for 9 days, then fed *ad libitum* for 6 days, and then wheel-running activity was assessed during 48 hours of food deprivation that began at either ZT3 (Fig. 1A, B) or ZT12 (Fig. 1C, D). When food was removed at ZT3 (Fig. 1B), minimal FAA was observed on the first day of food deprivation (72±55), but FAA increased significantly on the second day of fasting (2178±1261; *p* = 0.03). In contrast, FAA was robust on both the first (2187±3191) and second (5701±7547) days of food deprivation when food was removed at ZT12 (*p* = 0.37). It is possible that mice that began fasting at ZT3 were not “hungry” enough at ZT6 to display FAA on the first day of food deprivation. In contrast, mice that began fasting at ZT12 had 18 hours to become “hungry” and therefore displayed clear FAA at ZT6 the following day. Furthermore, FAA was observed on the second day of food deprivation in both conditions, suggesting that a certain period of food deprivation must be experienced before FAA is fully expressed. Since FAA was apparent on both days of fasting when food was removed at ZT12, we began fasting mice 18 hours before the time of previously scheduled food availability for all subsequent food deprivation experiments.

### BMAL1-deficient mice have robust FAA in the light-dark cycle

**Figure 2:**
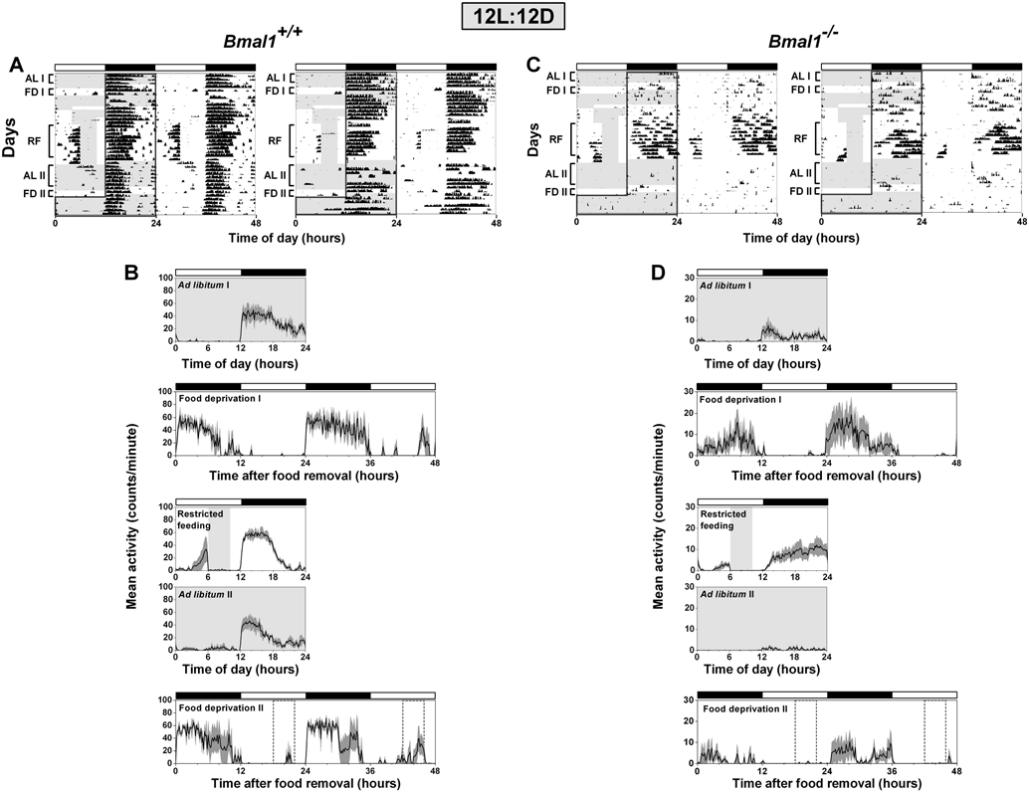
Food anticipatory activity in BMAL1-deficient mice in 12L:12D. Representative double-plotted actograms (A, C) and group average activity profiles (B, D) of *Bmal1* ^+/+^ mice (A, B; n = 3) and *Bmal1* ^−/−^ mice (C, D; n = 7) in 12L:12D. The time when food was available is indicated by light gray shading in the activity profiles and on the left half of each actogram. The light-dark cycle is indicated by the white and black bars, respectively. On the left half of each actogram, the dark black line outlines the time of darkness. The black traces in the group average activity profiles represent the mean number of wheel revolutions (in counts/minute) plotted in 10-minute bins relative to the light-dark cycle where ZT0 is lights on and ZT12 is lights off. The SEM is shown in dark gray shading in each activity profile. AL I, FD I, RF, AL II, and FD II labels of the actograms (A, C) indicate the days used to generate the mean activity profiles *ad libitum* I, food deprivation I, restricted feeding, *ad libitum* II, and food deprivation II, respectively (B, D).

**Figure 3:**
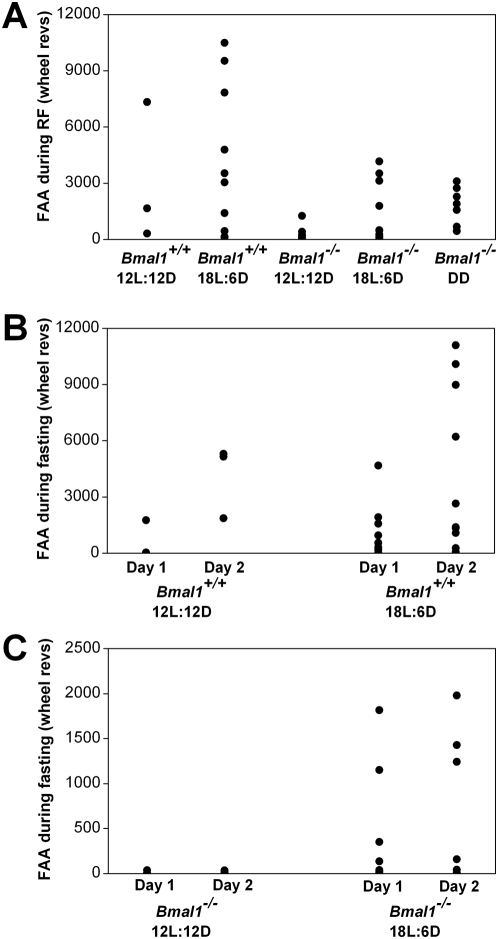
FAA from individual wildtype and BMAL1-deficient mice during restricted feeding and food deprivation. FAA during restricted feeding (RF; A) of individual mice in 12L:12D, 18L:6D, and DD was determined by totaling the number of wheel revolutions per minute from 4 hours before feeding time to the end of feeding time (total of 8 hours). FAA for each mouse was averaged over 9 days of restricted feeding. FAA during fasting for *Bmal1* ^+/+^ (B) and *Bmal1* ^−/−^ (C) mice was defined as the total number of wheel revolutions per minute from 4 hours before feeding time to the end of previous feeding time (total of 8 hours). Wheel-running FAA for each mouse was determined separately for the first (Day 1) or second (Day 2) day of food deprivation.

A previous study reported that *Bmal1^−/−^* mice did not display FAA and instead slept or were in torpor during restricted feeding [31]. In contrast, by gradually reducing food availability and by placing food pellets on the bottom of the cage, we found that most *Bmal1^−/−^* mice were active and healthy during restricted feeding [39]. We first performed restricted feeding in 12L:12D, a lighting condition that is typically used for studies in mice (Fig. 2). During 4-hour restricted feeding, FAA that began 2 to 4 hours before food was provided was observed in both *Bmal1^+/+^* (3101±3725) and *Bmal1^−/−^* (368±455) mice in 12L:12D (RF in Fig. 2A, C; Restricted feeding in Fig. 2B, D; FAA from individual mice shown in Fig. 3A).

During the succeeding 6 days of *ad libitum* feeding in 12L:12D, *Bmal1^+/+^* and *Bmal1^−/−^* mice had minimal daytime wheel-running activity (*Ad libitum* II in Fig. 2B, D). Mice were then fasted for 48 hours starting at ZT12. Similar to our control experiments (Fig. 1), group mean activity profiles from *Bmal1^+/+^* mice showed that mice were active during the time of prior food availability on the first (601±1005) and second (4104±1941) days of food deprivation (Fig. 2B; FAA from individual mice shown in Fig. 3B). In contrast, daytime activity was minimal or absent in *Bmal1^−/−^* mice on the first (9±13) and second (13±13) days of food deprivation in 12L:12D (Fig. 2D; FAA from individual mice shown in Fig. 3C).

**Figure 4:**
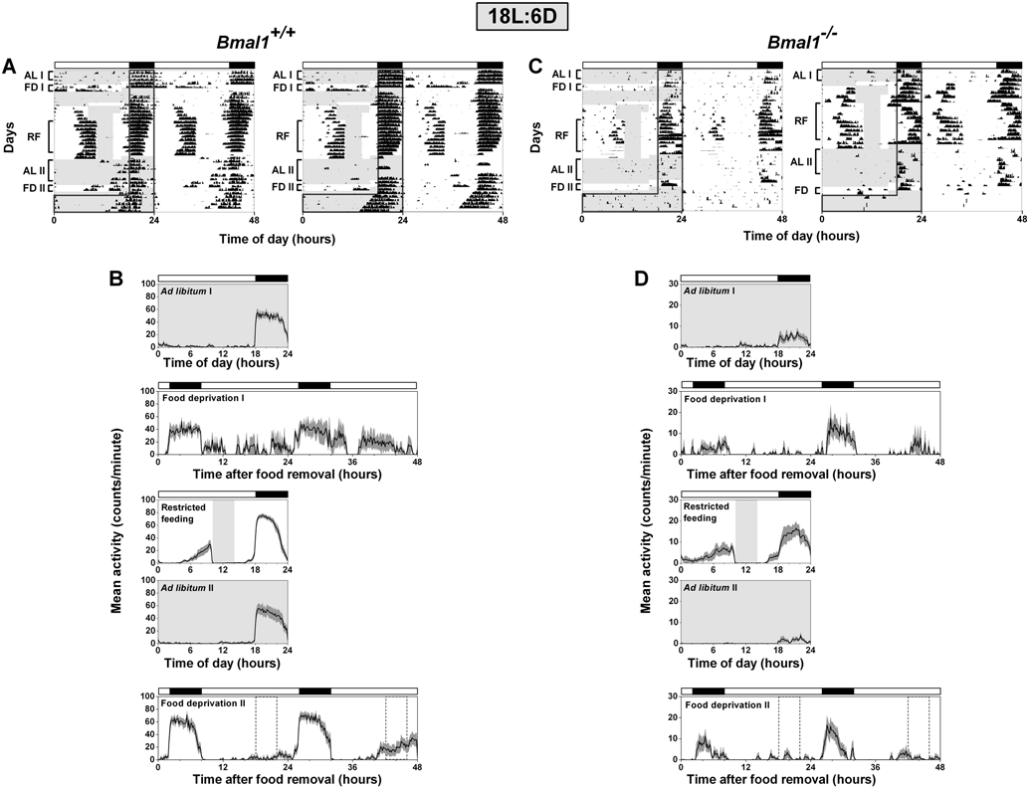
Food anticipatory activity in BMAL1-deficient mice in 18L:6D. Representative double-plotted actograms (A, C) and group average activity profiles (B, D) of *Bmal1* ^+/+^ mice (A, B; n = 10) and *Bmal1* ^−/−^ mice (C, D; n = 10) in 18L:6D. The time when food was available is indicated by light gray shading in the activity profiles and on the left half of each actogram. The light-dark cycle is indicated by the white and black bars, respectively. On the left half of each actogram, the dark black line outlines the time of darkness. The black traces in the group average activity profiles represent the mean number of wheel revolutions (in counts/minute) plotted in 10-minute bins relative to the light-dark cycle where ZT0 is lights on and ZT12 is lights off. The SEM is shown in dark gray shading in each activity profile. AL I, FD I, RF, AL II, and FD II labels of the actograms (A, C) indicate the days used to generate the mean activity profiles *ad libitum* I, food deprivation I, restricted feeding, *ad libitum* II, and food deprivation II, respectively (B, D). Food deprivation before restricted feeding (FD 1 or Food deprivation I) was performed in only 3 *Bmal1* ^+/+^ mice and 4 *Bmal1* ^−/−^ mice. All other mean activity profiles were generated from 10 mice of each genotype.

To determine if the lighting conditions could affect the expression of FAA in *Bmal1^−/−^* mice, we performed restricted feeding in 18L:6D (Fig. 4). During 4-hour restricted feeding in 18L:6D, robust FAA was observed in *Bmal1^+/+^* (4133±3922) and *Bmal1^−/−^* (1457±1576) mice (RF in Fig. 4A, C; Restricted feeding in 4B, D; FAA from individual mice shown in Fig. 3A). FAA during restricted feeding in *Bmal1^+/+^* (*p* = 0.69) and *Bmal1^−/−^* (*p* = 0.13) mice did not differ between 12L:12D and 18L:6D (Fig. 3A).

During *ad libitum* feeding following restricted food availability, daytime wheel-running activity was nearly abolished in *Bmal1^+/+^* and *Bmal1^−/−^* mice in 18L:6D (AL II in Fig. 4A, C; *Ad libitum* II in 4B, D). Mice were then fasted for 48 hours starting at ZT16. Group average activity profiles (Fig. 4B) and plots of FAA for individual mice (Fig. 3B) showed that FAA on day 1 (*p* = 0.69) and day 2 (*p* = 0.81) of food deprivation in *Bmal1^+/+^* mice did not differ between 12L:12D and 18L:6D. In contrast, wheel-running activity during food deprivation in *Bmal1^−/−^* mice differed greatly in 12L:12D and 18L:6D. While minimal wheel-running activity was observed in all *Bmal1^−/−^* mice during food deprivation in 12L:12D (Fig. 2C, D; FAA from individual mice shown in Fig. 3C), we observed FAA on both the first (392±651) and second (541±782) days of food deprivation in several *Bmal1^−/−^* mice in 18L:6D (Fig. 4C, D; FAA from individual mice shown in Fig. 3C). While FAA on day 1 (*p* = 0.09) and day 2 (*p* = 0.27) of food deprivation in *Bmal1^−/−^* mice did not significantly differ between 12L:12D and 18L:6D, examination of FAA in individual *Bmal1^−/−^* mice (Fig. 3C) showed that FAA during food deprivation was more prominent in 18L:6D than in 12L:12D. Notably, the pattern of wheel-running activity was similar during food deprivation before and after restricted feeding in 12L:12D (Fig. 2) and 18L:6D (Fig. 4).

Restricted feeding and food deprivation also affected nocturnal wheel running activity in *Bmal1^−/−^* mice. Similar to Bunger et al. [20], we found that *Bmal1^−/−^* mice (204±178) had lower average levels of nocturnal wheel-running activity than *Bmal1^+/+^* mice (2318±433) during *ad libitum* feeding in 12L:12D (AL I in Fig. 2A, C; *Ad libitum* I in 2B, D; *p*<0.001). Nocturnal activity during 4-hour restricted feeding (558±420) increased in *Bmal1^−/−^* mice compared to the antecedent period of *ad libitum* feeding (204±178; *Ad libitum* I vs. Restricted feeding in Fig. 2D; *p* = 0.07) in 12L:12D. The elevated nocturnal activity observed during restricted feeding decreased significantly during the successive period of *ad libitum* feeding (44±57; *Ad libitum* II vs. Restricted feeding in Fig. 2D; *p* = 0.02). Like 12L:12D, nocturnal activity in *Bmal1^−/−^* mice in 18L:6D increased during restricted feeding (450±290) compared to *ad libitum* feeding before (159±141; *Ad libitum* I in Fig. 4D; *p* = 0.01) and after food restriction (57±51; *Ad libitum* II in Fig. 4D; *p*<0.001). Nocturnal wheel-running activity did not differ between *ad libitum* and restricted feeding in *Bmal1^+/+^* mice in 12L:12D (Fig. 2A, 2B; *p* = 0.14) and 18L:6D (Fig. 4A, 4B; *p* = 0.32).

### BMAL1-deficient mice anticipate food availability in constant darkness

**Figure 5:**
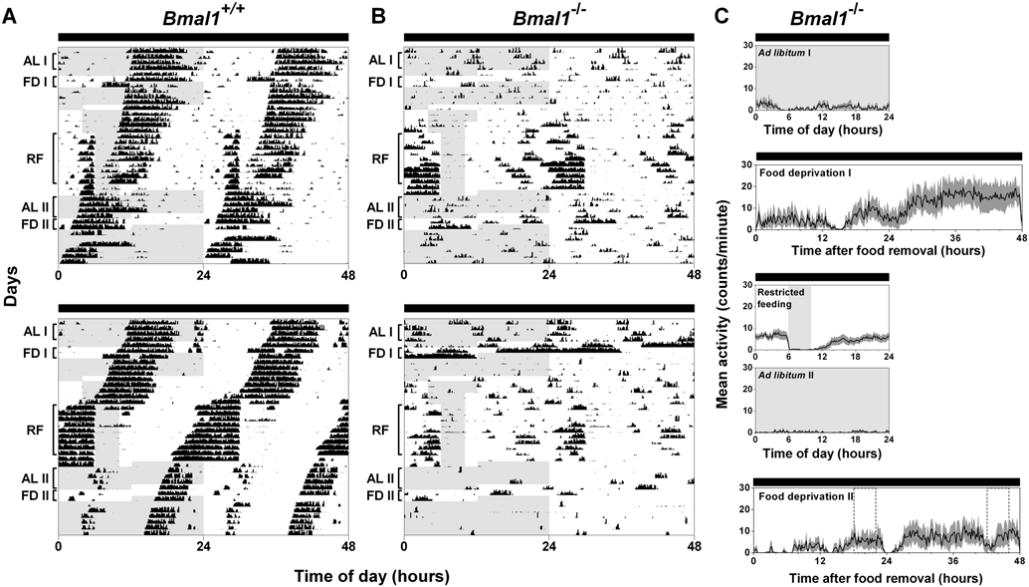
BMAL1-deficient mice display robust wheel-running activity prior to food availability in constant darkness (DD). Representative double-plotted actograms of wheel-running activity of *Bmal1* ^+/+^ (A; n = 6) and *Bmal1* ^−/−^ (B; n = 8) mice in DD. Group mean activity profiles for *Bmal1* ^−/−^ mice (C) were generated by averaging the number of wheel revolutions per 10-minute bin (black line) and were plotted relative to local time (where time 0 was the time of lights on and time 12 was lights off in the light-dark cycle prior to releasing the mice into DD). The SEM, which represents the variability among mice, is shown in dark gray shading. The time when food was available is indicated by light gray shading in the activity profiles and on the left half of each actogram. AL I, FD I, RF, AL II, and FD II labels of the actograms (B) indicate the days used to generate the activity profiles *ad libitum* I, food deprivation I, restricted feeding, *ad libitum* II, and food deprivation II, respectively (C).

It is unlikely that visual cues could account for the expression of FAA in *Bmal1^−/−^* mice since they display FAA during food deprivation after restricted feeding (Fig. 3C, Fig. 4). However, to test this possibility we performed restricted feeding in DD. Depending on the timing of free-running activity in *Bmal1^+/+^* mice, FAA was sometimes distinct from nocturnal activity (Fig. 5A, top panel), while it merged with nocturnal activity in other mice (Fig. 5A, bottom panel). Robust FAA always occurred prior to the time of food availability in *Bmal1^−/−^* mice in DD (1649±1041; RF in Fig. 5B; Restricted feeding in 5C, FAA from individual mice shown in Fig. 3A).

When released into *ad libitum* feeding, nearly all wheel-running activity was abolished in *Bmal1^−/−^* mice in DD (AL II in Fig. 5B; *Ad libitum* II in 5C). In 12L:12D and 18L:6D, we fed *Bmal1^−/−^* mice *ad libitum* for 6 days, while we provided food *ad libitum* for only 3 days in DD. If the FEO free-runs during *ad libitum* feeding in DD, then FAA during food deprivation should occur earlier or later than the previous time of restricted feeding. To avoid a large displacement of FAA from the previously scheduled time of feeding, we fed mice *ad libitum* for only 3 days in DD.

In the subsequent 48 hours of food deprivation, the wheel-running activity of individual *Bmal1^−/−^* mice in DD was variable. The wheel-running activity of 1 *Bmal1^−/−^* mouse was indistinguishable from *ad libitum* feeding (data not shown), 5 mice displayed distinct ultradian patterns of activity (Fig. 5B), and 1 mouse had nearly continuous activity with few breaks every 24 hours (data not shown). Occasionally, bursts of activity by *Bmal1^−/−^* mice occurred at about the time of previous food availability (FD II in Fig. 5B; Food deprivation II in 5C). However, we could not conclude that activity occurred at the previously scheduled mealtime in *Bmal1^−/−^* mice in DD because the mice were generally hyperactive. In addition, the pattern of wheel-running activity of *Bmal1^−/−^* mice during fasting before and after restricted feeding in DD was similar. During 48 hours food deprivation before restricted feeding, the wheel-running activity of 1 *Bmal1^−/−^* mouse did not differ from the previous *ad libitum* period (Fig. 5B, top panel), 3 *Bmal1^−/−^* mice displayed a distinct ultradian pattern of activity (data not shown), and 3 *Bmal1^−/−^* mice showed nearly continuous activity with only 2 or 3 breaks in 24 hours (Fig. 5B, bottom panel). Increased activity may be a response to fasting in *Bmal1^−/−^* mice in DD since total wheel-running activity increased during fasting before restricted feeding (1208±1079) compared to the antecedent period of *ad libitum* feeding (206±191; *p* = 0.05). Total activity was also elevated during food deprivation after restricted feeding (670±734) and did not differ from activity during fasting before restricted feeding (*p* = 0.30). Elevated wheel-running activity induced by food deprivation disappeared upon return to *ad libitum* feeding (Fig. 5A, B).

## Discussion

A previous study implicated *Bmal1* as an essential component of the FEO [31]. As previously reported, we hypothesize that that the failure to observe FAA in *Bmal1^−/−^* mice by Fuller et al. [31] can be attributed to the restricted feeding protocol used in that study [39]. In contrast to rats, access to food in mice should be gradually reduced over several days to prevent excess weight loss. In addition, though *Bmal1^−/−^* mice are healthy during *ad libitum* feeding when food is placed only on the wire top, we found that *Bmal1^−/−^* mice became almost completely inactive during restricted feeding, a condition that was reversed when we placed food on the floor of the cage [39]. While Fuller et al. did not report the number of *Bmal1^−/−^* mice investigated nor their mortality rate, food availability was abruptly reduced to 4 hours a day [31]. Furthermore, Fuller et al. had to physically arouse *Bmal1^−/−^* mice so that they would eat during the restricted feeding protocol [31]. While it was not explicitly stated by Fuller et al. [31] we assume that food was only provided on the cage top, which may have accounted for the lethargy of the *Bmal1^−/−^* mice. We predict that FAA would have been observed in *Bmal1^−/−^* mice in their study if food availability had been gradually reduced over several days and if food had been placed on the bottom of the cage.

Current and previous studies of FAA in *Bmal1^−/−^* mice during restricted feeding and food deprivation suggest that the light-dark cycle plays an important role in molding the expression of FAA [39]. FAA during restricted feeding is evident in both 12L:12D and 18L:6D. The mechanism for FAA expression in the light-dark cycle in *Bmal1^−/−^* mice could rely on an LEO with residual function. While the LEO is disabled *Bmal1^−/−^* mice in DD, as evidenced by arrhythmic locomotor activity and endogenous *Per1* and *Per2* gene expression in the SCN [20] and by arrhythmic *Per2* enhancer-driven luminescence in cultured SCN explants [40], it may be functional in the light-dark cycle. For example, in the light-dark cycle, wheel-running behavior in *Bmal1^−/−^* mice occurs more often during the dark than during the light phase [20] suggesting that the LEO may respond to light even if the oscillator is not fully functional. It is unclear whether the LEO is arrhythmic in *Bmal1^−/−^* mice in the light-dark cycle since circadian gene expression has not been assessed under these conditions. However, the expression of FAA in *Bmal1^−/−^* mice should not be influenced by the LEO or by masking in DD when the LEO is disabled. It is noteworthy then that we observe robust FAA in *Bmal1^−/−^* mice during restricted feeding in DD.

In 18L:6D FAA reappears in some mice at the time of previous entrainment to food availability when *Bmal1^−/−^* mice are food deprived. In contrast, we could not clearly distinguish FAA during food deprivation in 12L:12D. These findings show that experimental conditions, such as photoperiod, must be optimized during restricted feeding in circadian mutant mice. Interestingly, the activity of *Bmal1^−/−^* mice during food deprivation in DD does not resemble that in the light-dark cycle. Instead, nearly continuous or robust ultradian wheel-running activity is induced by food deprivation in DD. Sometimes activity occurs at the approximate time of food availability, but often does not. It is possible that the FEO is free-running in DD so that the timing of FAA is offset from the time of previous food availability. However, similar patterns of wheel-running activity are observed during food deprivation prior to restricted feeding and after restricted feeding, suggesting the possibility that this behavior is a direct response to lack of food, hunger, alteration in glucose levels, or other physiological changes associated with fasting. This process may take a couple of hours since there is very little wheel-running activity by *Bmal1^−/−^* mice for about 2 hours after food is removed during food deprivation before and after restricted feeding (time 12 of food deprivation I and II activity profiles).

Our findings that BMAL1-deficient mice display FAA during restricted feeding in the light-dark cycle raise a couple of possibilities regarding the nature of the FEO. First, the FEO may be a circadian oscillator that is composed of molecular timekeeping mechanisms that are unique from the LEO and cannot be explained by the current model of circadian transcriptional and translational feedback loop(s). Second, it is possible that the mechanism that generates FAA in BMAL1-deficient mice is not a self-sustaining oscillator, but instead it is an interval timer that measures the time between feeding and the phase of the LEO. If the latter is true, then FAA should disappear in *Bmal1^−/−^* mice in DD when the LEO is disabled. Our observation that *Bmal1^−/−^* mice display FAA during restricted feeding in DD suggests that the FAA can be expressed in the absence of the LEO if a daily feeding cycle is present. Findings from 30 years ago that SCN-lesioned rats anticipate food availability supported the hypothesis that the FEO must be a distinct circadian oscillator since it operated in the absence of the LEO. Since then numerous circadian oscillators located throughout the brain and body (i.e. retina, liver) have been described and additional oscillators will probably be identified in the future [41], [42]. Therefore, it is possible that FAA observed in *Bmal1^−/−^* mice could be the product of an interval timer or damped oscillator that is coupled to a circadian oscillator that is distinct from the SCN and does not require *Bmal1* for timekeeping. Finally, our finding that FAA in *Bmal1^−/−^* mice in DD does not regularly occur at the time of previous entrainment (or at a consistent phase relative to previous food availability) during food deprivation after restricted feeding, and that the pattern of wheel-running activity is similar during food deprivation before and after restricted feeding, raises the possibility that the mechanism for FAA is simply responding to the time of feeding in *Bmal1^−/−^* mice. In the absence of food, heightened activity occurs regardless of the previous feeding protocol. If this is the case, we cannot rule out that *Bmal1* is an important molecular component of the wildtype FEO, and that in the absence of *Bmal1*, the mechanism that controls the expression of FAA becomes an interval timer.

After 70 years of laboratory investigation of food entrainment in rodents [1], [2], the anatomical location and molecular timekeeping mechanism of the FEO remain elusive. The development and investigation of genetically altered mice have created new models for exploration of FAA. Our findings suggest that investigation of these genetically altered mouse models requires careful optimization of restricted feeding protocols. Furthermore, the expression of FAA may not be dependent on a circadian oscillator that requires BMAL1.

## Materials and Methods

### Animals

Experiments were conducted in accordance with the guidelines of the Institutional Animal Care and Use Committee at Vanderbilt University. *Bmal1* ^+/−^ mice [20] (generated by Dr. Chris Bradfield, University of WI) were obtained from Dr. Joseph Takahashi (Northwestern University) after they had been backcrossed for 9 generations (N9) with the C57BL/6J strain (Jackson Laboratory, Bar Harbor, ME). We performed additional backcrosses of *Bmal1* ^+/−^ mice with wildtype C57BL/6J mice so that mice were N13 to N15 when the experiments were performed. Heterozygous mice were then intercrossed to generate *Bmal1* ^+/+^ and *Bmal1* ^−/−^ mice. The mice were bred and group-housed in the Vanderbilt University animal facility in a 12h-light/12h-dark cycle (12L:12D) with food and water provided *ad libitum*. Male and female mice, aged 6 to 15 weeks at the beginning of the experiment, were used.

### Activity recording

For all experiments, naïve mice, which had never been exposed to an RF regimen, were used. Mice were singly housed in a cage (33×17×14 cm) with unlimited access to a running wheel (diameter: 11 cm) and water. The cages were placed in light-tight, ventilated boxes. The light intensity in each cage was approximately 350 lux. Wheel running activity was monitored by a micro-switch-activated signal using the ClockLab system (Actimetrics Inc.) and was collected by computer every minute.

### Restricted Feeding

Mice were fed LabDiet® 5001 Rodent chow (20% protein and 4.5% fat; Purina, Richmond, IN). In preliminary experiments, we found that *Bmal1^−/−^* mice became lethargic and nearly unresponsive to manual stimulation during the restricted feeding protocol when food was provided on the wire-top of the cage. By placing additional food on the bottom of the cage, we found that *Bmal1^−/−^* mice were healthy for the duration of the restricted feeding procedure. Therefore, for the experiments presented herein food was placed on the bottom of the cage for restricted feeding of *Bmal1^+/+^* and *Bmal1^−/−^*. Food was provided only on the wire-top for wildtype control mice (Fig. 1). Mice were allowed to eat as much as they desired during the time when food was available. When food was removed the light-tight box was opened and all food was removed from the wire-top and from the bottom of the cage and then the box was closed. During restricted feeding experiments performed in DD, an infrared viewer (FIND-R-SCOPE Infrared viewer, FJW Optical Systems, Inc., Palatine, IL) was used to add and remove food to cages so that mice were not exposed to visible light. During periods of *ad libitum* feeding and food deprivation, the light-tight boxes were not opened in order to avoid any external cues associated with food availability. During this time, the well-being of the mouse was monitored by assessing wheel-running data collected by the computer.

Wildtype mice were transferred from the breeding room to light-tight boxes (lights on from 06:00 to 18:00 local time). Following at least 3 days of *ad libitum* feeding, mice were food deprived for 24 hours (starting at ZT4, 10:00 local time) and then fed 8 h/day for 2 days and 6 h/day for 2 days (feeding started at ZT4). After this gradual reduction in food availability, mice were fed 4 h/day starting at ZT6 for at least 9 days, fed *ad libitum* for 6 days, and then food deprived for 48 hours starting at ZT12. Some WT mice were fasted for 48 hours starting at ZT3.

For experiments assessing wheel-running activity in *Bmal1^+/+^* and *Bmal1^−/−^* mice in 12L:12D, mice were transferred from the breeding room to light-tight boxes (lights on from 06:00 to 18:00 local time). Following at least 3 days of *ad libitum* feeding, mice were food deprived for 48 hours starting at ZT12. For experiments performed in 18L:6D, *Bmal1^+/+^* and *Bmal1^−/−^* mice were transferred from the breeding room to light-tight boxes (lights on 02:00 to 20:00 local time) and maintained in the new LD cycle for at least 1 week with *ad libitum* access to food and then food deprived for 24 or 48 hours starting at ZT16. For experiments assessing wheel-running activity in DD, mice were transferred from the breeding room (lights on 06:00 to 18:00 local time) to light-tight boxes and maintained in DD for at least 6 days with food provided *ad libitum*, and then were fasted for 48 hours starting at 18:00 local time.

Following 48 hours of fasting, all mice were fed *ad libitum* for 3 days, and then fed 8 h/day for 2 days and 6 h/day for 2 days (feeding started at ZT4, ZT6, or 10:00 CST in 12L:12D, 18L:6D, or DD, respectively). Then, mice were fed 4 h/day (starting at ZT6, ZT8, or 12:00 local time in 12L:12D. 18L:6D, or DD, respectively) for 10 days. In the light-dark cycle, mice were fed *ad libitum* for 6 days and then food deprived for 48 hours (starting at ZT12 or ZT16 in 12L:12D or 18L:6D, respectively). In DD, mice were fed *ad libitum* for 3 days and then food deprived for 48 hours (starting at 18:00 local time). Since it is possible that the FEO could free-run (with an unknown period) in DD during *ad libitum* feeding, we fed *ad libitum* for only 3 days to minimize the change in the phase of FAA when mice were fasted.

### Analysis

ClockLab software was used for behavioral analysis. Group average activity profiles were generated by averaging the counts per 10-minute bin for all mice in each group. The SEM in the mean activity profile graphs represents the variability among the mice in the group. FAA during restricted feeding was defined as the number of wheel revolutions per minute from 4 hours before feeding time to the end of feeding time (total of 8 hours). FAA for each mouse was averaged over 9 days of restricted feeding. Then, the mean FAA during restricted feeding was calculated by averaging the FAA for all mice. FAA during food deprivation was defined as the total number of wheel revolutions per minute from 4 hours before feeding time to the end of previous feeding time (total of 8 hours). Wheel-running FAA for each mouse was determined separately for the first or second day of food deprivation and the mean FAA was calculated from averaging the mice in each group. Nocturnal activity per day was determined by totaling the number of wheel revolutions during the dark portion of the LD cycle (ZT12 to ZT24 for 12L:12D and ZT18 to ZT24 for 18L:6D). The reported value of nocturnal activity represents the mean nocturnal activity averaged over 3 days for ALI, 6 days for ALII, and 9 days for restricted feeding. Total activity per day during *ad libitum* feeding was determined by measuring the total number of wheel revolutions for the entire 24-hour day. Then, mean total activity was calculated by averaging activity over 3 days of *ad libitum* feeding or 2 days of food deprivation.

### Statistics

Data (FAA during restricted feeding and food deprivation) are presented as the mean±standard deviation. Data were analyzed by *t* tests, except when data was not normally distributed or variances were not homogeneous. In these cases, the Mann-Whitney Rank Sum test was used.
